# Differential Polarization Nonlinear Optical Microscopy with Adaptive Optics Controlled Multiplexed Beams

**DOI:** 10.3390/ijms140918520

**Published:** 2013-09-09

**Authors:** Masood Samim, Daaf Sandkuijl, Ian Tretyakov, Richard Cisek, Virginijus Barzda

**Affiliations:** 1 Department of Physics and Institute for Optical Sciences, University of Toronto, 60 St. George Street, Toronto, ON M5S 1A7, Canada; E-Mails: masood.samim@utoronto.ca (M.S.); d.sandkuijl@utoronto.ca (D.S.); richard.cisek@utoronto.ca (R.C.); 2 Department of Chemical and Physical Sciences, University of Toronto Mississauga, 3359 Mississauga Road North, Mississauga, ON L5L 1C6, Canada; E-Mail: ian.tretyakov@mail.utoronto.ca

**Keywords:** differential microscopy, nonlinear microscopy, multiharmonic generation, genetic algorithms, adaptive optics

## Abstract

Differential polarization nonlinear optical microscopy has the potential to become an indispensable tool for structural investigations of ordered biological assemblies and microcrystalline aggregates. Their microscopic organization can be probed through fast and sensitive measurements of nonlinear optical signal anisotropy, which can be achieved with microscopic spatial resolution by using time-multiplexed pulsed laser beams with perpendicular polarization orientations and photon-counting detection electronics for signal demultiplexing. In addition, deformable membrane mirrors can be used to correct for optical aberrations in the microscope and simultaneously optimize beam overlap using a genetic algorithm. The beam overlap can be achieved with better accuracy than diffraction limited point-spread function, which allows to perform polarization-resolved measurements on the pixel-by-pixel basis. We describe a newly developed differential polarization microscope and present applications of the differential microscopy technique for structural studies of collagen and cellulose. Both, second harmonic generation, and fluorescence-detected nonlinear absorption anisotropy are used in these investigations. It is shown that the orientation and structural properties of the fibers in biological tissue can be deduced and that the orientation of fluorescent molecules (Congo Red), which label the fibers, can be determined. Differential polarization microscopy sidesteps common issues such as photobleaching and sample movement. Due to tens of megahertz alternating polarization of excitation pulses fast data acquisition can be conveniently applied to measure changes in the nonlinear signal anisotropy in dynamically changing *in vivo* structures.

## 1. Introduction

Biological samples often contain highly ordered molecular structures and microcrystalline aggregates that play important structural and physiological roles. Examples of these structures are ordered protein assemblies, such as collagen fibers and myosin filaments in striated muscle, as well as polysaccharide structures, such as starch granules and cellulose fibers. These structures usually have large birefringence and may exhibit nonlinear optical anisotropy. Therefore, measurement of different polarization parameters can elucidate the organization of molecules in these microcrystalline aggregates.

Polarization microscopy has been conveniently used to observe birefringent structures, such as starch granules, since the mid-19th century [1,2]. Sensitive measurements of differential polarizations can be performed on a pixel-by-pixel basis by combining an optical modulator of polarization and a lock-in amplifier in a laser-scanning microscope [3]. For example, fast beam polarization modulation has been used to image linear and circular dichroism of individual chloroplasts, revealing the chiral structure of the grana [4]. While birefringence and dichroism can be conveniently investigated with polarization microscopy, for thicker samples, where scattering becomes an issue, confocal laser scanning polarization microscopy can be used. The differential polarization technique has been employed in this context for measuring fluorescence detected linear and circular dichroism, anisotropy of fluorescence, as well as the degree of polarization of fluorescence [3,5–7].

Highly ordered structures also often exhibit signal anisotropy in the nonlinear optical regime. Various imaging modalities including multiphoton excitation fluorescence (MPF) [8], second harmonic generation (SHG) [9], and third harmonic generation (THG) [10] have been used in nonlinear confocal microscopy. Polarization measurements with a nonlinear microscope can be performed by sequentially imaging the sample at different polarizations [11], or by fast pulse-by-pulse polarization modulation. A novel nonlinear microscope system based on imaging with alternating polarization multiplexed laser beams has recently been demonstrated [12]. Images acquired during the same scan with the multiplexed beams were recorded simultaneously, detecting forward and epi-SHG in starch granules [12], as well as forward THG and epi-SHG in crystalline cellulose [13]. This means four different images were recorded with one raster scan. Multibeam scanning is very beneficial for imaging biological samples that undergo rapid structural changes or photobleaching. Recently, multifocal nonlinear microscopy has been reviewed [14–16].

In this paper, we demonstrate a new differential polarization microscopy setup with separate adaptive optics (AO) for each of the beams. The adaptive optics mitigate the problem of beam overlap, which is usually required for a multibeam system. This is especially important and difficult if multiplexed beam differential polarization microscopy is to be used for super-resolution investigations. In addition, adaptive optics can compensate for optical aberrations of the microscope components, and thereby improve its optical resolution [17–19]. For instance, deformable membrane mirrors (DMMs) have been successfully employed to optimize the point spread function (PSF) for SHG and THG in microscopes with single beam excitation [17,20]. The PSF improvement is especially effective for multiphoton processes, as the signal intensity increases nonlinearly with tighter focusing. AO can also be used for steering excitation beams. Both of these functions can be employed simultaneously in a differential multibeam microscopy setup for PSF optimization and two-beam overlap to ensure that the same voxel volume is excited in the sample by both beams.

In this article, we present a multibeam differential polarization microscopy setup and provide details on the implementation of the deformable mirrors as well as their performance in overlapping the two excitation beams, while simultaneously correcting for optical aberrations in the setup. Several applications of differential polarization microscopy are demonstrated, including SHG anisotropy measurements of collagen and cellulose fibers as well as fluorescence-detected nonlinear absorption anisotropy measurements of Congo Red-labeled cellulose. We show that differential polarization microscopy with multiplexed beams is a powerful technique for structural investigations of molecular orientations in ordered structures and sidesteps common issues such as photobleaching and sample movement artifacts in polarization measurements.

## 2. Differential Microscopy Setups

### 2.1. Principles of Differential Microscopy

**Figure 1:**
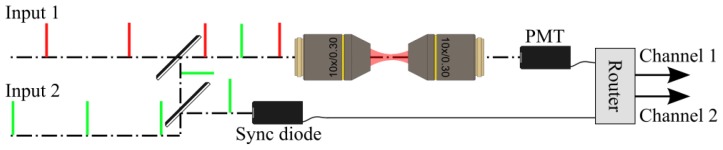
Schematics of differential microscopy. Two excitation beams are multiplexed, and a synchronization signal is derived from one of the beams. The sample is subjected to the multiplexed excitation, and the signal from the PMT is separated by a router containing fast electronics.

A differential microscopy setup can be divided into three functional parts: the laser-beam multiplexing unit, the microscope setup, and the signal-demultiplexing unit (Figure 1). While the microscope setup is very similar to other multiphoton laser scanning microscopes, the laser multiplexing and the signal demultiplexing units are novel additions. The schematics in Figure 1 show two synchronized pulsed laser beams that are combined so that their pulses are staggered in time. The sample is then subjected to the combined excitation beam. The signal generated from the sample is detected by a photon-counting detector (photomultiplier tube, (PMT) in Figure 1). A photodiode is used to provide a synchronization signal to the router, which sorts the signal pulses from the PMT into respective channels. The signal from the sample can be generated with linear or nonlinear excitation, as long as the excitation is performed by a pulsed laser source.

The excitation beams can differ in any of their characteristics: for instance, they can have different polarizations, or different wavelengths. More than two beams can be combined as well, as long as the inter-pulse spacing time is longer than the time window, which contains the signal from one of the beams. This limitation is mostly important in the case of fluorescence imaging where the signal is detected in a time window of up to tens of nanoseconds.

The laser pulses can originate from the same laser source, or from several synchronized lasers. The most straightforward implementation uses a single laser, a beam splitter and a delay line to generate multiple synchronized excitation beams [12]. This becomes cumbersome when the repetition rate of the laser source is less than a few tens of a megahertz. Multiple output couplers can be used in a long-cavity laser to achieve convenient pulse train delay and staggering of the excitation beams [21]. Previous implementations have shown up to six staggered output beams from a single laser cavity [22,23]. Combining the laser beams in differential polarization microscopy is most easily achieved with a polarizing cube beam splitter, which also ensures that the excitation polarizations are perpendicular to each other.

**Figure 2:**
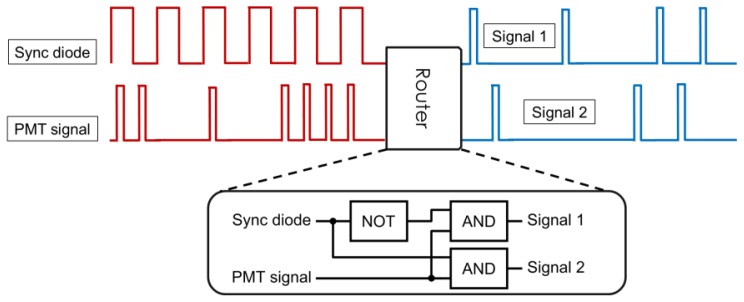
Schematics of signal demultiplexing. The PMT signal is sorted into demultiplexed channels for each beam based on the signal from the synchronization diode. The router can be implemented directly in programmable counting cards, or as a separate unit consisting of fast electronic logic gates (inset).

Generally, signal demultiplexing is performed using fast electronics (Figure 2). Some counting cards commonly used in microscopy can conveniently perform routing on-board [22], and when FPGA boards are used for signal counting it is relatively straight-forward to implement routing using an additional synchronization diode input [12,23]. Microscopes without these specialized counting electronics can still be adapted for differential microscopy by including a simple router based on fast logic gates as shown in Figure 2 (inset).

### 2.2. Implementation of the Differential Polarization Microscope

**Figure 3:**
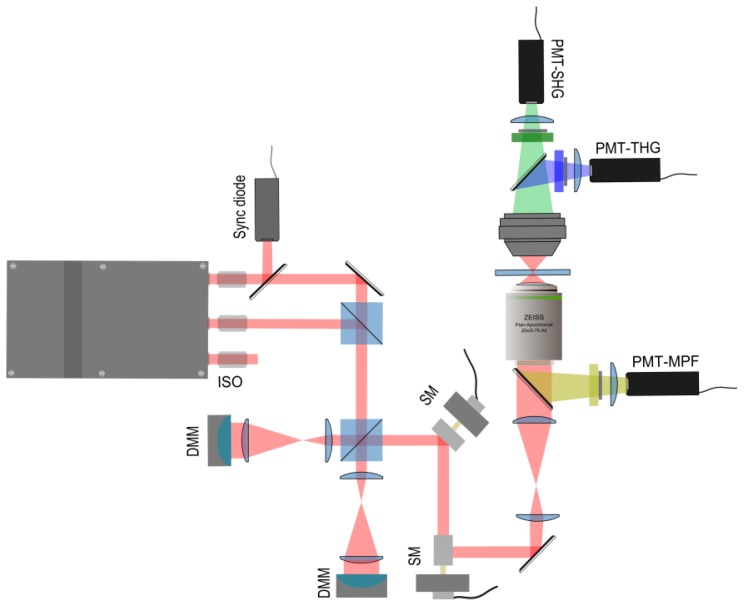
Schematics of the multimodal differential nonlinear microscope. Two Yb:KGW laser beams are coupled into the microscope for multiplexed imaging, and deformable membrane mirrors are used to shape the wavefronts of each beam. The multiplexed beam is reflected from scanning mirrors and relayed to the excitation objective. SHG and THG signals are collected in the forward direction, and the fluorescence signal is detected in the epi-direction using PMT detectors. PMT—photomultiplier tube, ISO—optical isolator, DMM—deformable membrane mirror, SM—canning mirror.

The outline of our custom-built multibeam nonlinear optical microscope setup is presented in Figure 3 [24]. Two orthogonally polarized infrared (1028 nm) beams from a home-built Yb: KGW laser are coupled into the microscope [21,22]. The laser has a repetition rate of 14.3 MHz, 430 fs pulse duration, and the beams originate from opposite ends of the laser cavity. The two excitation beams are combined using a polarizing cube beam splitter. Hence, the multiplexed beam has alternating pulses with perpendicular linear polarizations and a temporal spacing of about 35 ns between them [22]. Another polarizing cube splits the multiplexed beam and directs the polarization components towards two 39-actuator deformable membrane mirrors (DMMs, Flexible Optical BV, Delft, The Netherlands). The DMMs control the wavefront of each beam independently with up to 1 kHz operating frequency. The high operating frequency is particularly important for fast steering of the beams and for rapid point-spread function optimization in the microscope. The beams are recombined and directed to the resonant (15.5 kHz) and galvanometric (60–400 Hz) scanning mirrors (EOPC, Fresh Meadows, NY, USA) for lateral *x*- and *y*-scanning, respectively. The multiplexed scanning beam is relayed to the entrance aperture of the excitation objective, which has a numerical aperture (NA) of 0.75 (Zeiss, Jena, Germany). The second and third harmonic generation signals generated at the focus of the objective in the sample are collected in the forward direction by a custom-built UV-transmitting objective. The SHG and THG signals are separated by a dichroic mirror and detected with photomultiplier tubes (PerkinElmer, MP1343 RS CPM) [22,25]. For epi-detection measurements, we ensured that the dichroic mirror did not affect the excitation polarization significantly. Data acquisition, signal demultiplexing, and processing are done by an X5-210M FPGA board (Innovative Integration, Semi Valley, CA, USA). Data analysis is performed with custom MATLAB (The MathWorks, Natick, MA, USA) and LabVIEW programs (National Instruments, Austin, TX, USA).

### 2.3. Adaptive Optics for Differential Microscopy

The deformable mirrors can be conveniently used to optimize the PSF. The optimization can be achieved by maximizing the THG signal intensity from an air-glass interface of a coverslip [20]. The THG intensity at the interface can be approximated as follows:

(1) $$I(b)\propto {\left(\frac{1}{b}\right)}^{2}{\left|\int_{-\infty }^{z_{0}}\frac{e^{i\Delta kz^{\prime }}}{{\left(1+2iz^{\prime }/b\right)}^{2}}dz^{\prime }\right|}^{2}$$

where *z*′ is the axial coordinate, *z*_0_ is the position of the interface, *b* is the confocal parameter to be optimized and *Δk* is the wavevector mismatch due to dispersion. The full-width-at-half-maximum (FWHM) of the THG intensity profile as a function of the axial coordinate of the interface *z*_0_ is an approximation of the axial THG PSF [25] and is directly related to the confocal parameter *b* of the excitation beam. Since Equation 1 is based on simple Gaussian excitation and nonlinear signal beams, it is only an approximation when using an excitation objective with high numerical aperture.

**Figure 4:**
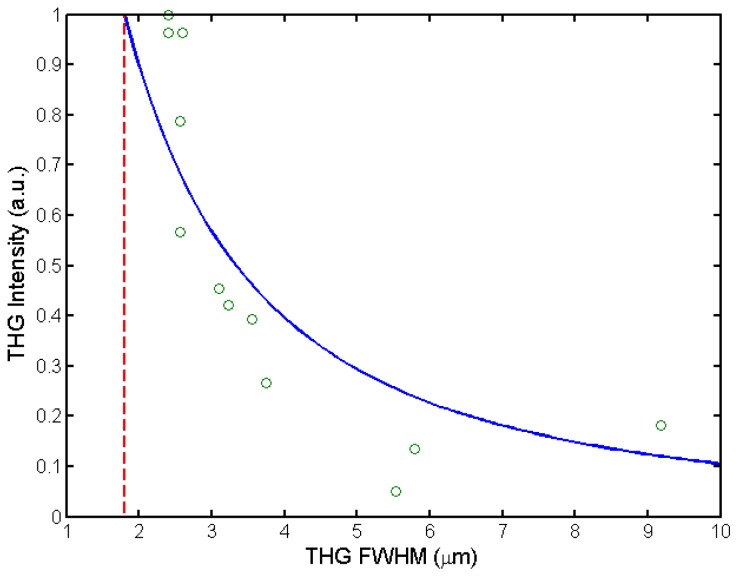
THG signal intensity dependence on the full width at half maximum (FWHM) of the axial THG PSF. THG signal is generated across a glass-air interface of a microscope coverslip (open circles) and the FWHM of the axial PSF follows the theoretical curve (Equation 1, solid line; *R*^2^*= 0.82*). For our excitation objective (0.75 NA, λ = 1028 nm), the theoretical minimum limit of the axial THG PSF is 1.8 μm (dashed line) [25].

As shown in Figure 4, the THG intensity from the interface increases with improvements in the PSF, and the experimental data fits well with Equation 1. In our nonlinear microscope, a minimum axial THG PSF of 2.0 ± 0.2 μm, which is close to the theoretical diffraction limit of 1.8 μm [25], can be easily achieved using the DMM (0.75 NA excitation objective, λ = 1028 nm).

#### Genetic Algorithm for Multibeam Control in Differential Microscopy

In addition to optimizing the PSF, it is also beneficial to use AO for beam steering in differential microscopy, since complete beam overlap allows for pixel-by-pixel comparison of the experimental data and ensures near-simultaneous excitation of a voxel by both excitation beams. Both goals can be achieved simultaneously using genetic algorithms (GAs). A GA is an evolutionary process whereby individuals' genes are mutated (and/or recombined and crossed over), and their relative fitness are computed and ranked. The gene combinations with the highest fitness are then selected as a basis for the next generation [26]. Previously, GAs were developed for improving imaging conditions in a single-beam microscope [27]. Usually, the nonlinear signal intensity from the sample is chosen as the fitness function, *i.e*., the parameter to be optimized during evolution [27,28] (see also Figure 4).

For the multibeam microscope, in order to also overlap the beams, the fitness function is modified to include a parameter based on the beam separation distance, *D*, where the beam position is calculated as an intensity-weighted average (centroid) of the image produced by each beam. The modified fitness function *F*_2beams_ is then defined as follows:

(2) $$F_{2beams}={wf}_{D}+(1-w)f_{I},\text{where }f_{D}=\frac{D_{\text{max}}-D}{D_{\text{max}}-D_{\text{min}}},f_{I}=\frac{I}{I_{\text{max}}}$$

where *f*_D_ is the normalized distance fitness parameter and *D*_max_ and *D*_min_ are the maximum and minimum distances obtained within each generation. Similarly, *f*_I_ is the normalized intensity contribution to the overall fitness function, and it depends on the intensity *I* generated from the sample by the steered beam. *I*_max_ is the maximum intensity obtained for the generation in question. The weighting-factor *w* ranges from zero to one and determines the contribution to the fitness function of the distance parameter relative to the intensity parameter. Practically, the value of *w* expresses the relative preference of the GA for maximizing the intensity *vs.* minimizing the distance during the optimization procedure.

**Figure 5:**
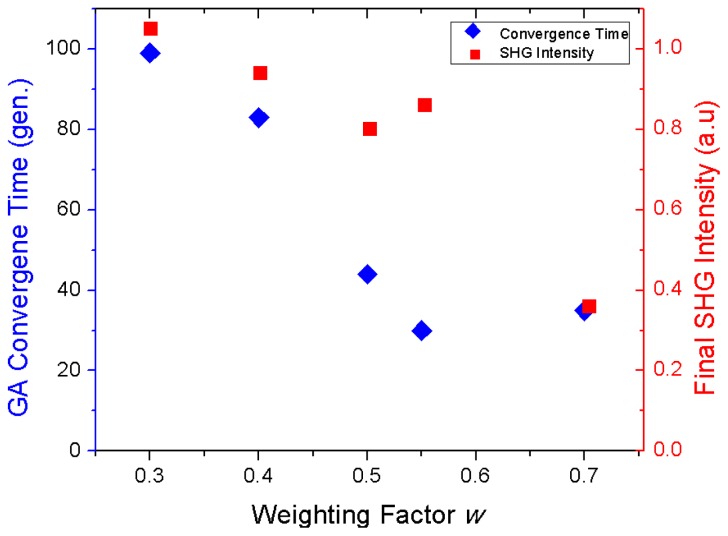
Dependence of the two GA optimization criteria on the value of the weighting factor *w*. The convergence time (blue diamonds, left *y*-axis) and final SHG intensity (red squares, right *y*-axis) are shown as a function of *w* for an initial separation distance of 6.3 ± 0.8 pixels (1.9 ± 0.2 μm). The optimum value for *w* appears to be around 0.55.

We set the GA to terminate when the mirror shape achieves a separation distance of less than 0.1 pixel (<30 nm) between the two excitation-beams. Therefore, two criteria are important for evaluating the optimal parameter *w*: (i) the number of generations necessary to reach the convergence, and (ii) the nonlinear signal intensity at the final position. How these optimization criteria depend on the value of *w* (Equation 2) is shown in Figure 5. It can be seen that higher values of *w* lead to shorter convergence times, as expected. On the other hand, higher values of *w* result in a lower final SHG intensity. Given this trade-off, the optimum value for *w* appears to be around 0.55.

The extent of initial beam separation that can be minimized by the positioning process is limited by the stroke range of the DMM electrostatic actuators. In our microscope, up to 4 μm separation distance between the centroids of the images of the two beams was successfully minimized with the GA control of one DMM (0.75 NA air objective). Employing a parallel GA for the second DMM doubles the initial separation distance that can be compensated.

High overlapping precision (<30 nm) becomes effortlessly accessible by using DMMs. In addition, DMMs driven by the GA correct aberrations introduced by the overlapping procedure, which is highly beneficial in nonlinear optical microscopy due to the nonlinear relationship between the signal intensity and the excitation beam wavefront quality. Thus, the overlapping procedure above becomes a necessary step for differential microscopy, where it must be ensured that the images from the multiplexed beam can be contrasted on a pixel-by-pixel basis. Such an overlap provides confidence in ratiometric studies of intensities from different polarizations as demonstrated in the Application section. The high accuracy overlap also allows using multiplexed beam differential polarization microscopy for super-resolution imaging applications, such as stochastic optical reconstruction microscopy (STORM) and photo-activated localization microscopy (PALM) [29,30].

## 3. Applications

Differential polarization microscopy can be applied for imaging with many different nonlinear image contrast mechanisms. In this section, we explore the scope of possible applications by highlighting SHG and fluorescence-detected absorption anisotropy.

### 3.1. Differential Polarization SHG Microscopy of Collagen Fibers

Polarization SHG from collagen fibers, an important biological structure, is regularly used to extract components of the second order polarizability tensor [11,31]. Most importantly, the ratio of tensor components $\rho =\chi_{zzz}^{\left(2\right)}/\chi_{zxx}^{\left(2\right)}$ has been linked to several characteristics of the collagen fibers, such as the protein amino acid composition and the structural organization of fibrils at several length scales [11,31,32]. Clearly, an accurate determination of the tensor components ratio has significant biomedical implications.

Commonly, ρ is determined by measuring the SHG intensity for many input polarization angles sequentially and fitting the polarization-dependent SHG intensity [11,31]. Instead, differential microscopy can be used to determine ρ in a single measurement by using multiplexed excitation beams polarized parallel and perpendicular to the collagen fiber orientation. The nonlinear susceptibility ratio ρ can then be immediately extracted using the relation $\rho =\sqrt{I_{\left|\right|}/I_{⊥}}$, where *I*_||_ and *I*_⊥_ are SHG intensities, respectively, and obtained by parallel and perpendicular laser polarizations with respect to the fiber [33,34].

**Figure 6:**
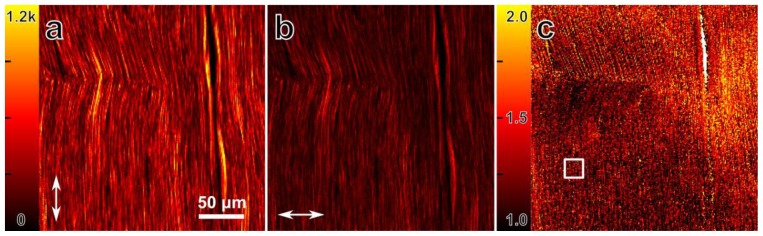
SHG polarization images from the stretched rat-tail tendon collagen sample used to determine ρ. (**a**,**b**) SHG images of the collagen sample with excitation polarization (indicated by the arrows) mostly parallel (**a**) and perpendicular; (**b**) to the fiber orientations; (**c**) The value of ρ for each pixel, calculated as $\rho =\sqrt{I_{\left|\right|}/I_{⊥}}$. The white square indicates the area used to determine a value of 1.36 ± 0.01 for ρ.

Figure 6 shows SHG signal from the stretched rat-tail tendon collagen sample used for this experiment, with the multiplexed excitation polarizations parallel and perpendicular to the fiber orientation (Figure 6a,b). The collagen fibers are well aligned, which indicates that their susceptibility ratio should be close to 1.4 [34]. Figure 6c shows ρ for each pixel in the sample image. For example, the extracted ρ from the white square in Figure 6c is 1.36 ± 0.01, which is calculated directly from the near-simultaneous excitation differential polarization nonlinear microscopy images. The only requirement is to keep the orientation of collagen fibers parallel to the polarization of one of the two beams. If the orientation of the fiber deviates from the polarization orientation, then other nonlinear susceptibility tensor components start contributing to the ρ ratio [34].

This experiment shows that SHG differential polarization microscopy can be used to extract quantitative information about the molecular orientation in the ordered tissue sample. Rapid measurements of ρ can be obtained, for example, in a specimen where collagen fibers are dynamically stretched during the experiment.

### 3.2. Absorption Anisotropy of Congo Red Stained Cellulose

**Figure 7:**
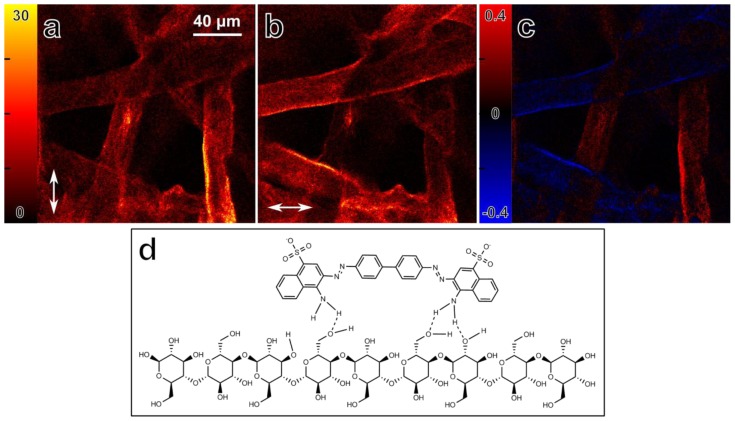
Fluorescence detected nonlinear absorption anisotropy of Congo Red molecules bound to a filter paper (cellulose). (**a**,**b**) Fluorescence intensity images, obtained simultaneously with different laser polarizations. The arrows indicate the polarization direction; (**c**) Image of fluorescence detected absorption anisotropy, defined as *r* = (*I_V_* − *I_H_*) / (*I_V_* + *I_H_*)_max_. Maximum absorption anisotropy occurs when one of the polarizations is parallel to the cellulose fibers; (**d**) Binding of Congo Red (top) to a cellulose molecule. Due to the symmetry of the Congo Red molecule, and many possible hydrogen bonding sites between cellulose and Congo Red, the diphenol backbone of the Congo Red molecule is likely to be parallel to the cellulose fiber orientation. Possible hydrogen bonds are indicated with dashed lines.

Absorption anisotropy within biological samples can reveal information about alignment and organization of molecules at the microscopic level. The absorption anisotropy is conveniently detected in the microscope using fluorescence signal. Differential microscopy can be used to measure fluorescence-detected absorption anisotropy in a single measurement, since excitation beams with orthogonal polarizations can be used almost simultaneously [22]. Figure 7a,b shows fluorescence intensity images from two orthogonal excitation polarizations, and Figure 7c shows the absorption anisotropy image from a sample consisting of Congo Red bound to cellulose fibers within a filter paper. The absorption anisotropy is defined here as *r* = (*I_V_* − *I_H_*) / (*I_V_* + *I_H_*)_max_, where the subscript refers to the vertical, V, and horizontal, H, orientation of the light polarization with respect to image.

Clearly, the nonlinear two-photon absorption is maximized when the polarization, as indicated by the arrow, is parallel to the cellulose fiber orientation. This is in line with the commonly accepted binding mechanism of Congo Red to cellulose, namely that the diphenol backbone of Congo Red is parallel to the cellulose fiber backbone (Figure 7d) due to the many possible hydrogen binding sites between the molecules [7,35,36].

Fluorescence is prone to photobleaching. Therefore, absorption anisotropy measurements by sequentially scanning the sample with two orthogonal laser polarizations may encounter major difficulties. Differential polarization microscopy with multiplexed beam excitation and electronic demultiplexing of the signal mitigates the photobleaching problem in the anisotropy measurements.

### 3.3. Multicontrast Differential Polarization Microscopy

**Figure 8:**
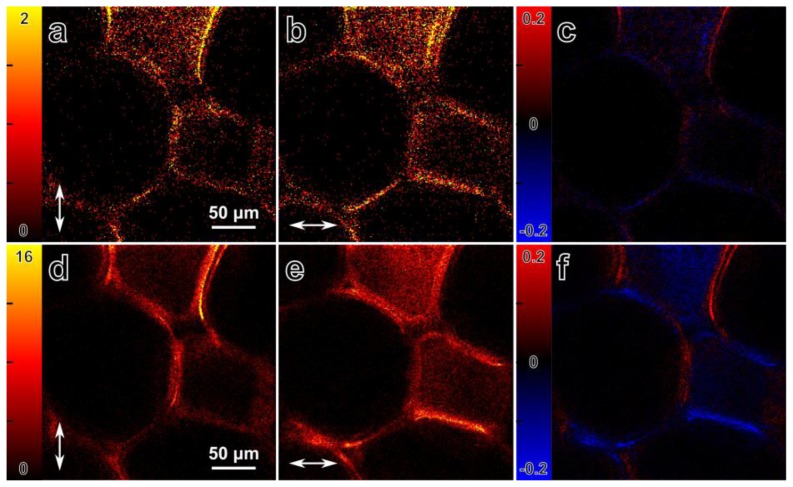
Multicontrast differential polarization imaging with SHG and fluorescence of Congo Red stained corn stalk tissue. (**a**,**b**) SHG intensity images, obtained simultaneously with orthogonal polarizations. The arrows indicate the polarization direction; (**c**) SHG anisotropy of the sample, defined as *r* = (*I_V_* − *I_H_*) / (*I_V_* + *I_H_*)_max_ ; (**d**,**e**) MPF intensity images obtained simultaneously with perpendicular polarizations. The arrows indicate the laser beam polarization direction; (**f**) Anisotropy of the fluorescence signal, defined as *r* = (*I_V_* − *I_H_*) / (*I_V_* + *I_H_*)_max_. Maximum signal occurs when the polarization is parallel to the cellulose fibers.

Differential polarization microscopy can also be used simultaneously with several image contrast mechanisms. SHG as well as fluorescence can be generated from cellulose labeled with Congo Red (Figure 8). Thus, the anisotropy of both signals can be used to probe the molecular organization of the cellulose fibers (by SHG) and the Congo Red molecules (through fluorescence) at the same time [12,37]. Figure 8 shows SHG intensity images (Figure 8a,b) and MPF images (Figure 8d,e) from a slice of dried corn stalk stained with Congo Red. The images are recorded with two multiplexed orthogonal laser polarizations using differential polarization microscopy. The panels in Figure 8c and Figure 8f show differential images, where the anisotropy is expressed as *r* = (*I_V_* − *I_H_*) / (*I_V_* + *I_H_*)_max_. From SHG images we can also estimate the second order susceptibility ratio ρ of the cellulose fibers, assuming that the fibers are oriented in the image plane and that the cylindrical symmetry applies to cellulose fibers. The areas were selected where the fiber direction was approximately parallel to the excitation polarization and the expression of $\rho =\sqrt{I_{\left|\right|}/I_{⊥}}$ is used to estimate ratio value, which is equal to 1.3 ± 0.1. However, due to the low signal level further measurements may be required for better estimation of the second order susceptibility ratio. The second order susceptibility ratio of the cellulose fiber may differ from the measured ratio depending on the fiber orientation out of image plane [34]. The fluorescence anisotropy (Figure 8f) confirms the results from subsection 3.2, showing maximum fluorescence signal from the Congo Red molecules when the polarization of the excitation beam is parallel to the cellulose fiber direction.

This study shows that both the molecular organization in ordered structures and the orientation of the marker-molecules within the labeled structure can be conveniently examined with differential polarization microscopy. It also demonstrates that quantitative information can be extracted from the sample by performing a single differential microscopy measurement.

### 3.4. High Accuracy Beam Overlapping for Differential Polarization Microscopy

**Figure 9:**
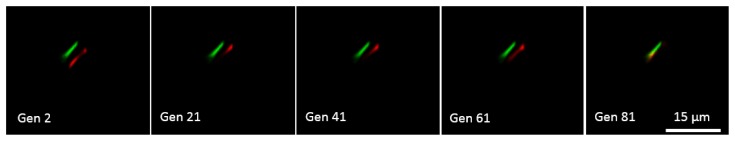
Montage (Supplementary information) of SHG from a single ZnSe nanowire generated by the two perpendicularly polarized multiplexed beams during the GA-assisted beam overlapping procedure. Green: image from the stationary reference beam; red: image from the beam subjected to the GA. The weighting factor *w* was increased to 0.5 at the 9th generation. The overlapping process converged after 81 generations, which took approximately 5.5 min.

A time-lapse of a two-beam overlapping procedure using the nonlinear differential polarization microscope is presented in Figure 9 (Supplementary information). The multiplexed laser pulses of the two excitation-beams were used to generate two distinct SHG images from a ZnSe nanowire (NW). The ZnSe NWs sample was ideal for overlapping because of the high SHG signal intensity, polarization-dependent SHG response and convenient size, with diameter ranging from 60 to 100 nm and length ranging from a few to tens of micrometers [38,39]. The two beams were initially displaced by ~3 μm, and we used the multiparameter fitness function (Equation 2) to overlap the images from the two excitation beams. Initially the signal intensity was maximized by using a weighting factor *w* of zero. The value of *w* was increased to 0.5 at the 9th generation to overlap the beams, which led to a complete overlap of the two images of the NW after about 5.5 min (Figure 9, Supplementary information).

The convergence time for achieving the high accuracy overlapping is acceptable for routine alignment of multibeam differential microscopy. Further improvements of the time constraint would benefit from advances in the field of adaptive optics including the directed search of mirrors shape space and adopting Zernike or Lukosz modes for controlling the shape of mirrors [40]. The overlapping precision shown above (<30 nm) is an indispensable tool for studies of, for instance, contracting muscle cells. Within a myocyte, a sarcomere length can be measured with 20 nm accuracy by determining the intensity centroids [41]. During muscle contraction, sarcomeres undergoing length changes exhibit different SHG intensity changes for different polarizations. The precision for differential polarization microscopy prescribed here allows for dynamically measuring the changes in SHG anisotropy, which can be related to structural changes of the myosin filaments in the sarcomeres [42].

## 4. Conclusions and Outlook

Differential polarization nonlinear optical microscopy is a very sensitive imaging technique capable of measuring anisotropy of nonlinear signals with photon counting sensitivity. The technique sidesteps common issues such as photobleaching and sample movement and allows for imaging of biological samples with multiple nonlinear image contrast mechanisms simultaneously, which means the technique can be used in many challenging applications, where weak polarization signals have to be measured or polarization anisotropy has to be obtained during movement of the sample. The applications of SHG and nonlinear absorption anisotropy presented in this study on collagen and cellulose examples show that orientational and structural properties of the fibers can be quickly and accurately obtained. The implementation of adaptive optics ensures high precision overlap of the two beams and allows pixel-by-pixel calculation of the measured anisotropy. Structural and accompanying polarization changes can be determined in dynamic samples. Therefore, time-multiplexed differential microscopy with photon counting detection and adaptive optics beam shaping is an attractive tool for dynamic imaging applications.

## Supplementary Files

Supplementary File 1 Supplementary (AVI, 10952 KB)
